# *Butia catarinensis* as a Sustainable Ingredient in Brazilian Mead: Chemical Properties, Volatile Composition, Sensory Profile and Consumer Acceptance

**DOI:** 10.1111/1750-3841.71007

**Published:** 2026-03-29

**Authors:** Rodrigo Ribeiro Arnt Sant'Ana, Ana Letícia Andrade Ferreira, Rafaela Diogo Silveira, Juliane Elisa Welke, Bruna Rafaela da Silva Monteiro Wanderley, Luciano Valdemiro Gonzaga, Adriane Costa dos Santos, Ana Carolina Oliveira Costa, Renata Dias de Mello Castanho de Amboni, Carlise Beddin Fritzen‐Freire

**Affiliations:** ^1^ Department of Food Science and Technology Federal University of Santa Catarina (UFSC) Florianópolis Brazil; ^2^ Food Science and Technology Institute Federal University of Rio Grande do Sul (UFRGS) Porto Alegre Brazil

**Keywords:** aromatic, CATA, GC‐MS, honey wine, jelly palm

## Abstract

Mead is a fermented alcoholic beverage traditionally produced with honey, water, and yeast, with possible variations through the addition of fruits or spices. *Butia catarinensis* is a native Brazilian fruit with high sensory appeal, traditionally used in cachaça but underexplored in fermented beverages. Therefore, this study aimed to evaluate the potential of butia‐matured mead by analyzing its physicochemical parameters, volatile profile, sensory characteristics and consumer acceptance. Three formulations were developed with different concentrations of butia during maturation (30 days at 4°C): traditional mead (TM—without butia), mead matured with 15% butia (w/w) (BM15), and mead matured with 30% butia (w/w) (BM30). The samples underwent physicochemical analyses, volatile organic compounds and sensory characterization using hedonic scale and check‐all‐that‐apply (CATA). Maturation with butia increased the acidity and pH of the samples while decreasing the content of reducing sugars. Malic acid was predominant in butia‐matured meads, whereas gluconic acid prevailed in traditional mead. Volatile profile revealed a distinct profile among the meads, with esters being the predominant class. Maturation with butia introduced specific compounds, such as ethyl decanoate and ethyl dodecanoate, not detected in traditional mead. CATA results indicated that TM was associated with honey‐related characteristics, BM30 with fruity attributes, and BM15 exhibited intermediate features, being the most accepted sample by consumers (86% of acceptance index). This study successfully demonstrates the potential of *Butia catarinensis* as an ingredient for the beverage industry, providing a viable strategy to produce differentiated meads while valorizing an underutilized Brazilian fruit.

**Practical Applications:**

This study provides a practical strategy for mead producers to create innovative beverages. The results show that maturing mead with 15% (w/w) *Butia catarinensis* produces a beverage with high consumer acceptance (86% index), successfully integrating fruity notes with the characteristic honey flavor of traditional mead. This process offers a way to diversify the mead market. Furthermore, using this underutilized fruit supports sustainable practices and adds value to Brazilian biodiversity. The check‐all‐that‐apply method is confirmed as an effective and accessible tool for the industry to guide product development based directly on consumer perception.

## 1 Introduction

Mead is a fermented alcoholic beverage produced using water, honey, and yeast (Wanderley et al., 2022). Beyond traditional mead, it is common to produce variations with the addition of fruits (melomel) or spices (metheglin), allowing the creation of differentiated products with high sensory value (Kawa‐Rygielska et al., 2019). Depending on the type of fruit or fruit juice added to the mead, the beverage receives specific designations, such as pyment (mead + grape), cyser (mead + apple), morat (mead + blackberry), among others (Sant'Ana et al., 2023). Although several studies have investigated fruit‐based meads (Adamenko et al., 2018, 2021; Chitarrini et al., 2020; Kawa‐Rygielska et al., 2019; Schwarz et al., 2022), research exploring the incorporation of underutilized Brazilian fruits remains limited (Amorim et al., 2018; Wanderley et al., 2022).

Brazil is recognized for its great biodiversity and numerous native fruits with attractive sensory characteristics, offering potential for enhancing mead with distinct flavors. One such species is butia (*Butia* spp.), a palm tree native to South America, distributed across Brazil, Argentina, Uruguay, and Paraguay (Boeing et al., 2020; Corrêa et al., 2009). The primary consumption forms of this fruit include fresh consumption, juices, sweets, and traditionally, butia‐matured cachaça (Büttow et al., 2009). However, due to its high perishability, butia remains underutilized, with consumption largely restricted to its natural growing regions and harvest seasons (Rockett et al., 2021). Butia contains important antioxidants such as ascorbic acid, beta‐carotene, and phenolic compounds (Barbosa et al., 2021). The fruit exhibits a color spectrum from yellow to orange‐reddish hues, an intense aroma combining fruity and citrus attributes, and a distinctive flavor that balances sweetness and acidity (Vinholes et al., 2018). Additionally, its pulp is succulent and fibrous (Teixeira et al., 2019), with the capacity to produce a pleasant and aromatic wine‐like beverage. Bernardi et al. (2014) analyzed the volatile composition of *Butia odorata* fermented beverages and identified various compounds characterized by floral and fruity aromas. These aromatic notes are also associated with higher aromatic quality in mead (Li and Sun, 2019), suggesting a promising combination between butia and the beverage.

One of the native butia species from the southern Brazilian coast and currently at risk of extinction is *B. catarinensis*. Its fruits are primarily harvested through sustainable wild collection in restinga vegetation (coastal shrubland areas), serving as an essential source of livelihood for low‐income traditional communities that use and commercialize them (Pedroso et al., 2023). Therefore, strategies for processing and commercialization, such as incorporating butia into beverages such as mead, could promote plant conservation, expand product availability across different regions and seasons, and support beekeeping through honey value addition. Furthermore, given the traditional consumption of butia‐matured cachaça in Brazil and the demonstrated consumer interest in butia‐flavored mead, as indicated by an unpublished consumer study, it is hypothesized that combining butia with another alcoholic beverage may result in a product with high consumer acceptability.

To assess the sensory characteristics, acceptability, and feasibility of a novel product, sensory analysis is a widely employed tool (Stone et al., 2020). In recent years, the check‐all‐that‐apply (CATA) test has gained prominence in food and beverage sensory research due to its simplicity and reliability (Ares and Jaeger, 2023; Lee et al., 2021; Manzatti et al., 2019). This method is used to describe products based on consumer perception, providing a detailed and easily reproducible sensory profile. CATA has been widely applied to assess sensory attributes in fermented beverages such as wine (Kemp et al., 2019; Manzatti et al., 2019), cider (Phetxumphou et al., 2020), sake (Lee et al., 2021), and traditional mead (Sant'Ana et al., 2025). However, no studies have yet applied CATA to evaluate the sensory characteristics of fruit meads, nor have any investigations analyzed the sensory profile of butia‐matured mead. Applying CATA in this context allows for a direct understanding of consumer perception, which, when combined with chemical data (volatile compounds and organic acids), can establish a comprehensive quality profile and uncover the links between the chemical composition and the sensory experience of the beverage. Therefore, this study aimed to evaluate the physicochemical parameters, volatile profile, and sensory attributes of meads matured with different concentrations of *Butia catarinensis*, using the CATA methodology to obtain a consumer‐based sensory characterization and to understand how chemical changes manifest sensorially.

## 2 Materials and Methods

**FIGURE 1:**
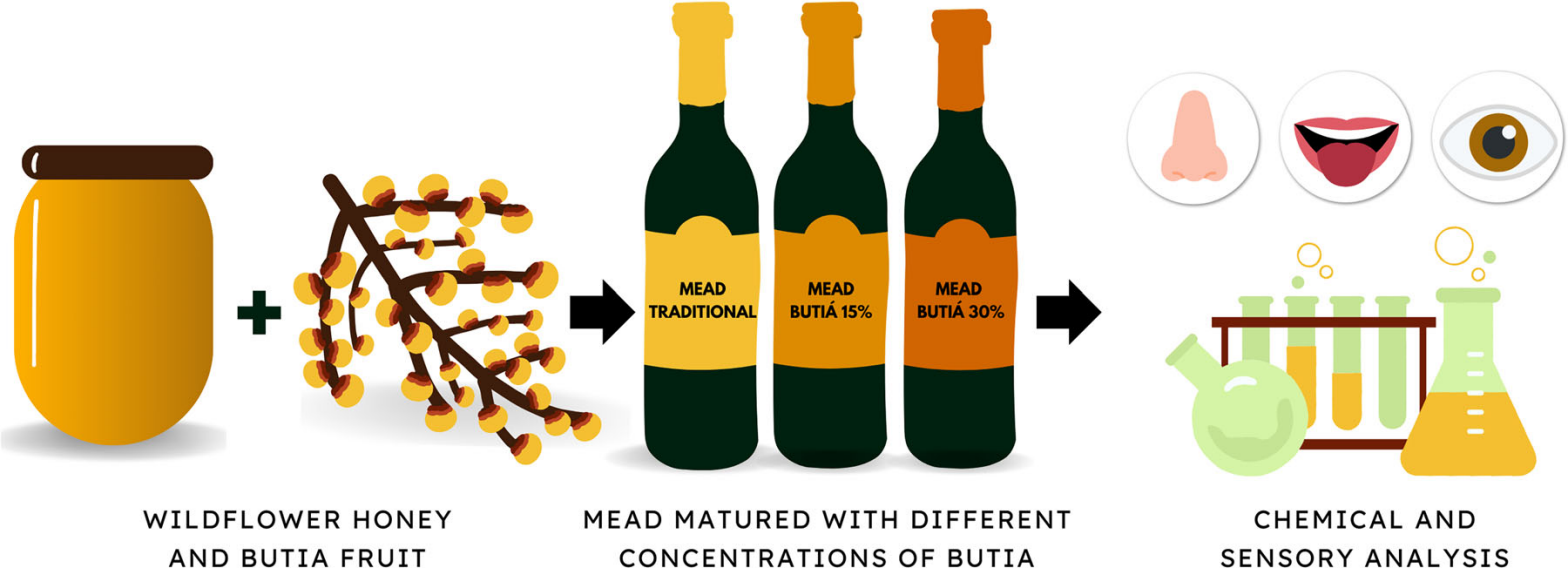
Production and characterization of mead matured with butia

The methodological flowchart of this study is presented in Figure 1. Three mead formulations were developed using different concentrations of butia during maturation. Physicochemical, volatile compounds and sensory analyses were conducted to characterize the products.

### 2.1 Reagents

Ultrapure water used for solution preparation was obtained through a Milli‐Q system (Bedford, USA). Potassium metabisulfite and sodium hydroxide were purchased from Neon Comercial (Suzano, Brazil). Analytical standards of organic acids (maleic, malonic, fumaric, tartaric, formic, citric, malic, glycolic, lactic, gluconic, succinic, glutaric, acetic, and propionic acids) and standards of organic volatile compounds (octanoic acid, 1‐hexanol, *cis*‐3‐hexen‐1‐ol, *cis*‐2‐hexen‐1‐ol, 2,3‐butanediol, phenylethyl alcohol, ethyl butanoate, ethyl hexanoate, ethyl octanoate, ethyl decanoate, α‐phellandrene, *p*‐cymene, *β*‐linalool, nerol, *α*‐pinene) were purchased from Sigma‐Aldrich (St. Louis, USA) and Aldrich (Steinheim, Germany), respectively.

### 2.2 Fermentation Process

Wildflower honey (Pró‐Apis, São Bonifácio, Brazil) from a certified supplier in Santa Catarina (Brazil) was used for the fermentation of the meads. The butia fruits (*Butia catarinensis*) were collected between January and March 2023 in the city of Imaruí, Santa Catarina, Brazil (28°16'37.3“S 48°45'50.4”W ‐ 55 m altitude), sanitized with sodium hypochlorite (100 mg/L), vacuum packed, and frozen (−20°C) until use.

The fermentation of the meads was carried out following the methodology of Wanderley et al. (2022), with modifications. The work was prepared by diluting honey in mineral water (Da Guarda, Tubarão, Brazil) to achieve a total soluble solids content of 22 ± 0.2 °Brix. Yeast *Saccharomyces bayanus* (Premier Blanc, Red Star, Belgium) (0.5 g/L), mead nutrient (Brewnutri‐Z, Angel, China) (10 mg/L), and potassium metabisulfite (50 mg/L) were added. The fermentation process was conducted in glass fermenters (Uniglass, Barra do Ribeiro, Brazil) with airlocks, in a BOD incubator (TE‐371, Tecnal, Brazil) at 22 ± 1°C. The fermentation was monitored by measuring the total soluble solids (TSS) every 4 days. Fermentation lasted 12 days until the mead reached 11 ± 0.2 °Brix. At this point, the mead underwent clarification, during which it was refrigerated at 4 ± 1°C for 48 h, then transferred and bottled in 750 mL glass bottles (Embavale, Rio Morto, Brazil). The maturation of the meads was performed in glass bottles for 30 days at 4 ± 1°C. During this stage, the whole butia fruits were added with skin, separated into different treatments: Traditional mead (TM), without butia; Mead matured with 15% butia (w/w) (BM15); and Mead matured with 30% butia (w/w) (BM30). The fruit concentration, maturation time, and method of addition were defined based on preliminary tests and traditional formulations of butia‐based beverages.

### 2.3 Physicochemical Analyses

All physicochemical analyses were performed in triplicate. The analyzed parameters in the meads were: total soluble solids (°Brix) measured with a digital refractometer (HI96801, Hanna Instruments, Barueri, Brazil); alcohol content (% v:v) using an ebulliometer (Linklab, São Paulo, Brazil) (OIV, 2012); pH and total acidity (mEq/L) measured with a pH meter (Tec‐7, Tecnal, Piracicaba, Brazil) at 25 ± 1°C; reducing sugars (g/L) by the Lane–Eynon method (Fehling), as described in the Manual of Analytical Norms of the Adolfo Lutz Institute (IAL, 2008).

### 2.4 Organic Acids

The profile of organic acids was determined using a capillary electrophoresis system (model 7100, Agilent Technologies, Santa Clara, California, USA) equipped with a diode‐array detector, following the methodology proposed by Brugnerotto et al. (2019), with modifications. The data were processed using the HP ChemStation software (rev A.06.01). The separations were carried out in a fused silica capillary (Polymicro Technologies, Phoenix, AZ) with dimensions of 60.5 cm total length, 52 cm effective length, 75 µm inner diameter, and 375 µm outer diameter. The samples were centrifuged at 10,000 rpm for 15 min (MiniSpin plus, Eppendorf AG, Hamburg, Germany), and the supernatants were filtered through a 0.45 µm filter. The filtrates were then diluted at a 1:10 ratio (v/v; sample: deionized water). The filtered samples were further diluted with the internal standard (fluoroacetic acid) in a 9:1 ratio (v/v, sample: internal standard) and injected into the CE‐DAD system. When necessary, additional dilutions of the filtrates were performed.

Quantification of organic acids followed the procedure described by Brugnerotto et al. (2019), which involved the use of calibration curves prepared in both standard solution and matrix‐matched solution. Seven equally spaced concentration levels (1.3–121 mg·L^−1^) were prepared in triplicate, and quantification was performed using the ratio between the peak area of each analyte and that of the internal standard (fluoroacetic acid), added to all samples and standards at a fixed concentration.

### 2.5 Instrumental Color

The instrumental color determination of the meads was performed using a colorimeter (Minolta Chroma Meter CR‐400, Minolta, Osaka, Japan), set to operate with a 10° observation angle and D65 illuminant. All analyses were performed in triplicate. The parameters were measured using the CIELab color scale, where the L* parameter indicates color variation from black to white (0–100), the a* parameter indicates variation from red (+a*) to green (−a*), and the b* parameter indicates variation from yellow (+b*) to blue (−b*). The C* parameter represents chromaticity (color saturation), and the h* is the hue angle (hue). The ΔE calculation (a parameter indicating color difference between samples) was also performed to confirm if the color differences observed were perceptible to the naked eye. For this, the following formula was used:

$$\Delta E=\sqrt{{\left(\Delta L^{*}\right)}^{2}+{\left(\Delta a^{*}\right)}^{2}+{\left(\Delta b^{*}\right)}^{2}}$$

### 2.6 Profile of Volatile Organic Compounds (VOCs)

#### 2.6.1 Extraction of Volatile Compounds by Headspace Solid‐Phase Microextraction (HS‐SPME)

The extraction of volatile organic compounds (VOCs) was performed using the headspace solid‐phase microextraction (HS‐SPME) technique. A 1 mL aliquot of each sample was transferred to a 20 mL glass headspace vial. To enhance the extraction efficiency through the salting‐out effect, sodium chloride (NaCl) was added to achieve a concentration of 30% (w/v). The vials were sealed and subsequently equilibrated in the sample heater at 45°C for 10 min before the fiber was exposed. The extraction was conducted for 45 min at a constant temperature of 45°C without sample agitation. A 2 cm fiber coated with a divinylbenzene/carboxen/polydimethylsiloxane (DVB/CAR/PDMS, 50/30 µm, Supelco, Bellefonte, PA, USA) stationary phase was used for sampling the headspace. After the extraction period, the fiber was thermally desorbed in the GC injection port for 5 min at 250°C. To eliminate any residual compounds and prevent carryover between runs, the fiber was routinely reconditioned for 5 min at 260°C prior to each new analysis. All samples were analyzed in triplicate.

#### 2.6.2 Gas Chromatography‐Mass Spectrometry Conditions

The separation and detection of the extracted volatile compounds were carried out using a Gas Chromatography system coupled to a quadrupole Mass Spectrometer (GC‐qMS) (QP‐2010, Shimadzu Corporation, Kyoto, Japan). The chromatographic separation was performed using a DB‐WAX capillary column (100% polyethylene glycol; 30 m × 0.25 mm × 0.25 µm film thickness) purchased from J&W Scientific (Folsom, CA, USA). The mass spectrometer was operated in electron ionization (EI) mode at 70 eV. The transfer line temperature was maintained at 250°C, and the ion source temperature was set at 230°C. Mass spectra were acquired in the range of 40 to 350 *m* ***/*** *z*. Volatile compounds were identified by comparing their mass spectra with the NIST library and by comparing their experimental linear retention indices with those reported in the literature for similar stationary phases. The co‐injection of the following analytical standards (Sigma‐Aldrich) was performed in DB‐WAX column: 1‐hexanol, *cis*‐3‐hexen‐1‐ol, *cis*‐2‐hexen‐1‐ol, 2,3‐butanediol, ethyl butanoate, ethyl hexanoate, ethyl octanoate, ethyl decanoate, octanoic acid, phenylethyl alcohol, α‐phellandrene, *p*‐cymene, *β*‐linalool, nerol, *α*‐pinene. A homologous series of *n*‐alkanes (C_7_–C_24_, Supelco) was analyzed under the same chromatographic conditions as the samples to calculate the experimental linear retention index (RI) for each detected compound, based on the method of Van Den Dool and Kratz (1963). Volatile compounds were tentatively identified when the difference between the experimental RI and the literature RI was less than 10 units.

#### 2.6.3 Quantification of Volatile Compounds

Quantification of VOCs was performed using a semiquantitative approach, following the principles adopted in previous HS‐SPME–GC/MS studies and adapted to the analytical conditions of this work (Welke et al., 2012). The area of the following internal standards (IS) was used to normalize the area of sample compounds according to chemical classes: C13‐norisoprenoids and terpenes (1,4‐cineole), acids (isobutyric acid), alcohols (3‐octanol), aldehydes and ketones (dodecane), ethyl and methyl esters (methyl nonanoate), and acetate esters (phenyl acetate). A mixed standard solution (10 mg/L) containing all analytical standards was prepared in ethanol, and 10 µL of this solution was added to each sample before HS‐SPME analysis.

A synthetic model matrix was prepared with similar ethanol content (10%), pH (3.5), and sugar concentration (23 g L^−1^ glucose) to the mead samples and was used for calibration in order to minimize matrix‐related extraction effects inherent to the HS‐SPME technique. External calibration curves were constructed using analytical standards (4‐ethyl‐phenol, hexanoic acid, decanoic acid, octanal, nerol, 3‐hexenol, benzaldehyde, 1‐hexanol, 1‐dodecanol, 2‐ethyl‐1‐hexanol, ethyl decanoate, and octanoic acid), selected as representative compounds for their respective chemical classes (purity greater than 98%, St. Louis, USA). Quantification was performed by normalizing each VOC peak area to the corresponding internal standard and applying the calibration curve of the chemically closest external standard. Because not all VOCs had an identical available analytical standard, the results represent semiquantitative concentrations, expressed in mg/L equivalents of the corresponding reference standard, as commonly employed in HS‐SPME–GC/MS studies.

### 2.7 Sensory Analysis

The sensory tests were conducted in a climate‐controlled room (22 ± 2°C) at the Department of Food Science and Technology of the Center of Agricultural Sciences (CCA) at the Federal University of Santa Catarina (UFSC) (Florianópolis, Brazil), a facility widely used for sensory evaluations at the institution. The environmental conditions were rigorously controlled, ensuring uniform lighting and minimizing external interference to guarantee the reliability of the participants' responses. The sensory analysis was approved by the Ethics Committee on Human Research (CEPSH) at UFSC (No. 71912223.5.0000.0121), in both ethical and methodological aspects, complying with the guidelines established by Resolution No. 466, dated December 12, 2012, of the National Health Council (BRASIL, 2012).

The participants in the sensory analysis (*n* = 102) were recruited through virtual and physical advertisements. Both men and women over 18 years old (the legal drinking age in Brazil) and regular consumers of alcoholic beverages participated in the research. The recruitment strategy included digital announcements on university social media pages and institutional mailing lists, in addition to posters and printed flyers distributed across the campus. The final consumer panel consisted of 57.8% women and 42.2% men, with ages ranging from 18 to 63 years (mean ≈27 years). Approximately 94% reported regular consumption of alcoholic beverages, and around 68% had previously consumed mead. Although a substantial portion of participants likely came from the university environment, this mixed recruitment approach resulted in a relatively heterogeneous consumer group.

The sensory analysis was conducted with 30 mL of each mead sample (10 ± 2°C), served monadically to the participants following a balanced presentation order. The samples were presented in transparent acrylic cups and coded with random three‐digit numbers. Participants were also provided with a glass of water and cream cracker biscuits to cleanse their palates between sample evaluations (Stone et al., 2020). The evaluation form provided to participants requested information on gender, age, whether they consumed fermented alcoholic beverages, and whether they had previously tried mead, in addition to the acceptability and CATA tests.

The hedonic test to evaluate the liking of the mead samples followed the methodology of Pascoal et al. (2017), with modifications. A nine‐point hedonic scale was used, anchored at the extremes as “(1) extremely disliked” and “(9) extremely liked,” assessing overall liking and by attributes (appearance, aroma, and flavor) of the samples. To verify the overall liking rate of the beverages, the acceptability index (AI) was calculated using the formula: AI = A*100/B, where A is the average score obtained in the test and B is the maximum score used for product evaluation (Dutcosky, 2013).

The CATA method was used to identify descriptive characteristics of the samples based on the consumers' perceptions. The questionnaire consisted of a list of 25 terms, including sensory descriptor attributes selected from previous studies that conducted sensory analysis on mead or wine (Lee et al., 2021; Manzatti et al., 2019; Senn et al., 2021; Starowicz and Granvogl, 2020; Sant'Ana et al., 2025), as well as terms adapted for this study with the help of a focus group consisting of six fermented beverage specialists. The presentation of the terms was randomized and balanced within each category for the different evaluators (Meyners and Castura, 2016). Participants were instructed to select the terms from the list that they considered appropriate to describe each mead sample. The 25 terms presented in the CATA questionnaire were grouped into the following categories: Appearance (yellow, golden, clear, cloudy), Aroma (sweet, honey, floral, fruity, citrus, alcoholic, white wine, butia), Mouthfeel (alcoholic, astringent, dry, tingling), and Taste/Flavor (sour, bitter, sweet, honey, floral, fruity, balanced, butia, butia cachaça).

### 2.8 Statistical Analysis

Analysis of variance (ANOVA) and Tukey's test were used to identify significant differences (*p* < 0.05) between the samples in the physicochemical analyses, volatile compounds and liking tests. The profile of organic acids in the samples was assessed through principal component analysis (PCA). The frequency data of the attributes for each sample obtained in the CATA test were tabulated in contingency tables, and the Cochran Q test was applied, followed by a post‐hoc Sheskin's critical difference test to identify significant differences (*p* < 0.05) between the frequencies of the attributes in each sample. Associations between the attributes that presented significant differences in CATA and the mead samples were assessed through Correspondence Analysis (CA). To identify how attributes influenced the acceptability of the samples, a penalty analysis was applied (Meyners and Castura, 2016). The statistical analysis was performed using the STATISTICA software version 7.0 (TIBCO Inc., Palo Alto, USA), except for PCA, CA, Cochran *Q* test, and penalty analysis, which were performed using XLSTAT software (Version 2021.1, New York, USA).

## 3 Results and Discussion

### 3.1 Physicochemical Characteristics

The physicochemical parameters of meads matured with different concentrations of butia are presented in Table 1. The alcohol content and total soluble solids (TSS) of the meads ranged from 10.15% to 10.40% and 10.73 to 10.87 °Brix, respectively, with no statistically significant differences (*p* > 0.05) between the samples. Wanderley et al. (2022) produced meads with different fruits and achieved alcohol content ranging from 11.60% to 12.30%, also observing no significant differences in this parameter compared to the traditional mead produced without fruit. The highest values of pH and total acidity (mEq/L) (*p* < 0.05) were observed in the BM30 sample, followed by the BM15 and TM samples, confirming that the addition of butia during the maturation stage of the beverage promoted an increase of approximately 38% in the total acidity of the BM30 sample compared to the TM sample. This increase can be justified by the composition of organic acids derived from the fruits (Antunes et al., 2024). Furthermore, among the butia species, the one used in this study (*Butia catarinensis*) has the highest total acidity, around 3.22% (expressed as citric acid) (Antunes et al., 2024; Rockett et al., 2021). The addition of fruits to mead was also studied by Chitarrini et al. (2020), who evaluated the effect of blackcurrant (*Ribes nigrum*) on the physicochemical parameters of the beverage, observing that the mead made with wildflower honey and fruit also had higher pH values and lower residual sugar concentrations than the meads without fruit, with no difference in alcohol content and TSS between the samples.

**TABLE 1:** Physicochemical parameters of the different meads.

Samples	Total soluble solids (°Brix)	pH	Total acidity (mEq/L)	Alcohol content (%)	Reducing sugars (g/L)
**TM**	10.73 ± 0.06^a^	3.43 ± 0.01^c^	48.41 ± 0.84^c^	10.15 ± 0.21^a^	31.94 ± 1.44^a^
**BM15**	10.83 ± 0.06^a^	3.52 ± 0.01^b^	56.47 ± 0.64^b^	10.20 ± 0.14^a^	21.57 ± 0.42^b^
**BM30**	10.87 ± 0.10^a^	3.58 ± 0.02^a^	67.24 ± 0.64^a^	10.40 ± 0.00^a^	18.29 ± 0.52^c^

Results expressed as mean ± standard deviation.

^a–c^Different lowercase letters in the same column indicate a significant difference (*p* < 0.05) between mead types for the same parameter.

TM: traditional mead; BM15: mead matured with 15% butia; BM30: mead matured with 30% butia.

The concentration of reducing sugars in the meads showed a significant difference (*p* < 0.05), with the highest concentration observed in the TM sample, followed by the BM15 and BM30 samples (Table 1). This decrease in the concentration of reducing sugars in the butia meads may be caused by the fact that butia has a high moisture content (>70%) (Pereira et al., 2022), which can lead to the dilution of the reducing sugars in the meads. Another possibility is that the fibers of butia might have absorbed some of the components present in the mead, as the fruit have a high fiber content (on average 30%) (Rockett et al., 2020). Vitorino‐Junior et al. (2024) produced meads with wildflower honey and observed reducing sugar contents ranging from 14.38 to 30.08 g/L, similar to those found in the meads of this study. Other studies also reported similar behaviors in terms of reducing sugar content in mead samples, where meads made with fruits and herbs (dandelion syrup, chokeberry, and grape seed addition) had lower sugar content compared to traditional mead (Kawa‐Rygielska et al., 2019). Thus, it was found that the variations in the physicochemical parameters of the mead samples are justified by the presence of butia in the beverages, as reported by other studies that produced mead with fruits (Chitarrini et al., 2020; Kawa‐Rygielska et al., 2019; Wanderley et al., 2022).

### 3.2 Organic Acids

The organic acids analysis revealed the influence of maturation with butia on the organic acid profile of the meads (Table 2). The predominant organic acid in the TM sample was gluconic acid, in agreement with the literature, which indicates the predominance of this acid in floral honeys (Brugnerotto et al., 2019), as well as in meads (Starowicz and Granvogl, 2020). No significant differences (*p* > 0.05) were observed in the gluconic and succinic acid contents between the different samples. The TM sample had the highest concentration of lactic acid (*p* < 0.05) compared to the meads containing butia. This organic acid, along with malic, acetic, and citric acids, is common in floral honeys, although in lower concentrations compared to gluconic acid (Bergamo et al., 2019; Seraglio et al., 2019).

**TABLE 2:** Concentration of organic acids (mg/L) in the meads.

Organic acid	TM	BM15	BM30
**Fumaric**	<0.72*	<0.72*	<0.72*
**Formic**	<0.70**	<0.70**	49.90±2.38
**Citric**	65.48 ± 1.16^c^	153.63 ± 7.49^b^	213.18 ± 8.85^a^
**Malic**	994.57 ± 38.19^c^	3856.13 ± 178.77^b^	5942.87 ± 79.18^a^
**Glycolic**	<0.09*	<0.09*	<0.09*
**Lactic**	124.53 ± 3.43^a^	84.46 ± 2.51^c^	95.97 ± 1.55^b^
**Gluconic**	3089.10 ± 103.97^a^	3153.88 ± 104.19^a^	3001.04 ± 59.93^a^
**Succinic**	1010.51 ± 19.40^a^	1016.29 ± 33.52^a^	1016.57 ± 8.84^a^
**Acetic**	524.34 ± 10.90^a^	507.17 ± 33.52^a^	410.82 ± 9.96^b^

Results expressed as mean ± standard deviation. Different letters in the same row indicate significant differences (p < 0.05) between samples according to Tukey's test.

TM: traditional mead; BM15: mead matured with 15% butia; BM30: mead matured with 30% butia.

*Minimum detection limit of the method.

**Minimum quantification limit of the method

Butia fruits have high acidity, resulting from a high concentration of organic acids, especially malic and citric acids (Hoffmann et al., 2017). This characteristic was also observed in the meads matured with butia, which showed malic acid as the main acid, with the BM30 sample exhibiting the highest concentration of this compound (*p* < 0.05). Wanderley et al. (2022) also reported that meads with native Brazilian fruits—feijoa (*Acca sellowiana*) and uvaia (*Eugenia pyriformis*)—had high levels of malic acid, which is one of the predominant acids in various types of fruit. Citric acid concentrations were also significantly higher in the BM30 and BM15 samples, with increases of approximately 225% and 134%, respectively, compared to the sample without butia (TM). These findings align with the literature and suggest that the addition of fruits may increase the citric acid content in meads, as observed by Švecová et al. (2015), where meads with cherry also exhibited high citric acid concentrations. Simão et al. (2023) highlight that the presence of citric acid in meads can improve the antioxidant activity and color retention of the beverage. Zhu et al. (2022) explain that citric and malic acids are the two main organic acids found in fruits, and are also present in significant amounts in butia (Hoffmann et al., 2017), which justifies the elevated presence of these compounds in the fruit‐matured mead samples. Finally, the BM30 sample showed the highest concentration of formic acid and the lowest concentration of acetic acid (*p* < 0.05) among the samples evaluated. The notable decrease in acetic acid in BM30 is a positive finding, as high concentrations of this acid are often associated with an undesirable vinegar‐like character in fermented beverages.

**FIGURE 2:**
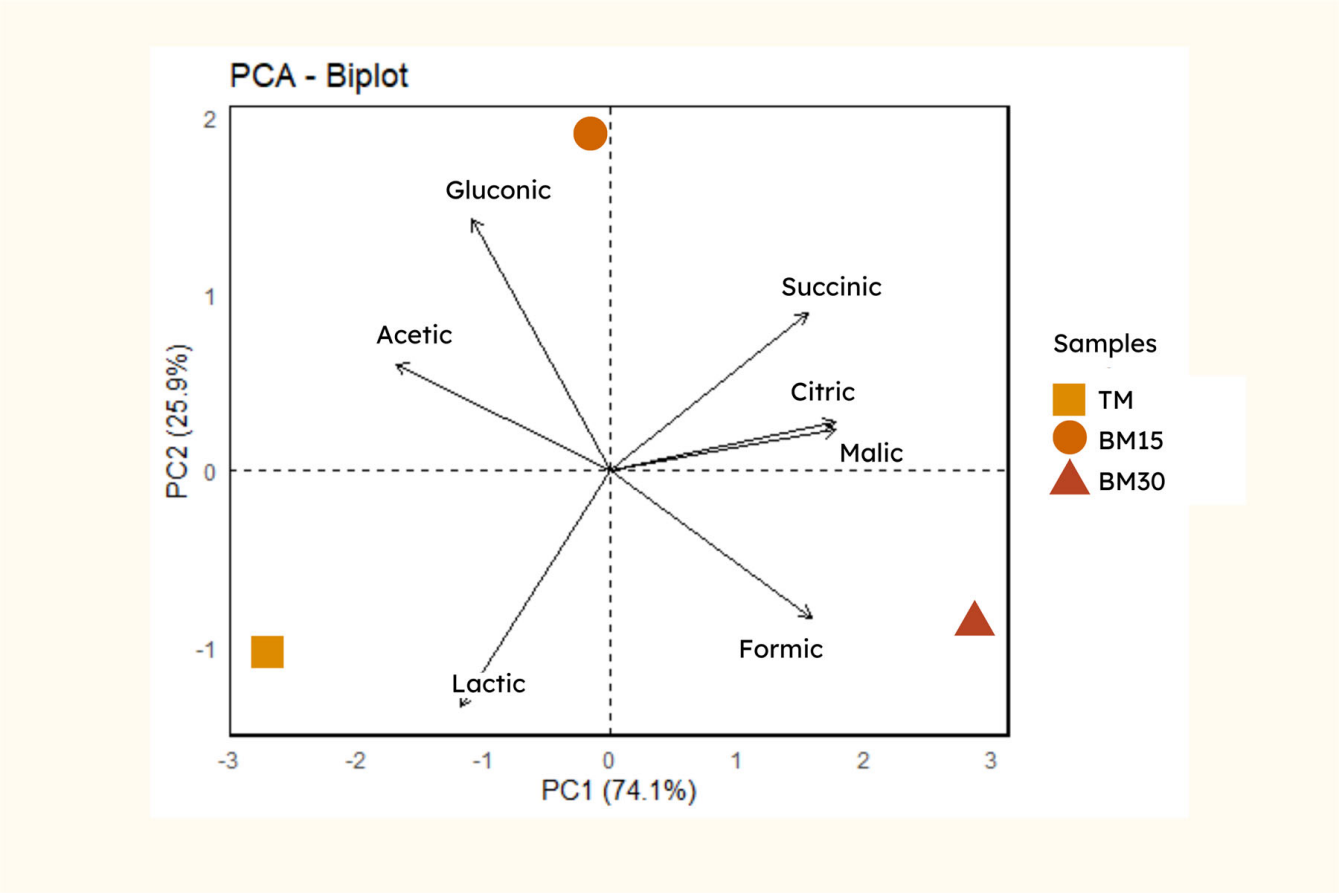
Principal component analysis of the organic acid profile. TM: traditional mead; BM15: mead matured with 15% butia; BM30: mead matured with 30% butia.

The principal component analysis (Figure 2) demonstrated the distribution of the TM, BM15, and BM30 samples based on their organic acid profiles. The PC1 axis explained 74.1% of the total variability, primarily differentiating the samples by the concentrations of acetic, gluconic, succinic, citric, and malic acids. The PC2 axis, responsible for 25.9% of the variability, contributed to an additional separation based on lactic and formic acids. The gluconic and acetic acids were positioned between the TM and BM15 samples (Figure 2), which is explained by the similar concentrations of these acids in both samples (Table 2). The BM30 sample, located in the lower right quadrant, showed an association with citric, succinic, and malic acids, as well as formic acid. In contrast, the TM sample, positioned in the lower left quadrant, was associated with lactic acid.

A more detailed inspection of the loading vectors further supports this interpretation: gluconic acid shows a strong positive contribution to PC2, consistent with the upper positioning of BM15; lactic acid loads negatively on PC2, matching the location of TM; and malic, citric, succinic, and formic acids contribute positively to PC1, explaining the separation of the butia‐matured samples along this axis. These loading patterns reinforce that the addition of butia shifts the acid profile in a structured manner, with each organic acid contributing differentially to the observed multivariate separation. Therefore, the PCA provided insight into how different organic acids contribute to the distinction of the mead samples matured with butia, highlighting the influence of fruit concentrations on organic acid profiles, which can directly impact the sensory perception of these beverages (Yan et al., 2024). Specifically, the clear separation along PC1 underscores a direct gradient from a “honey‐dominated” acid profile (TM) to a distinct “fruit‐dominated” profile (BM30), with BM15 occupying an intermediate and potentially more balanced position.

### 3.3 Instrumental Color

**FIGURE 3:**
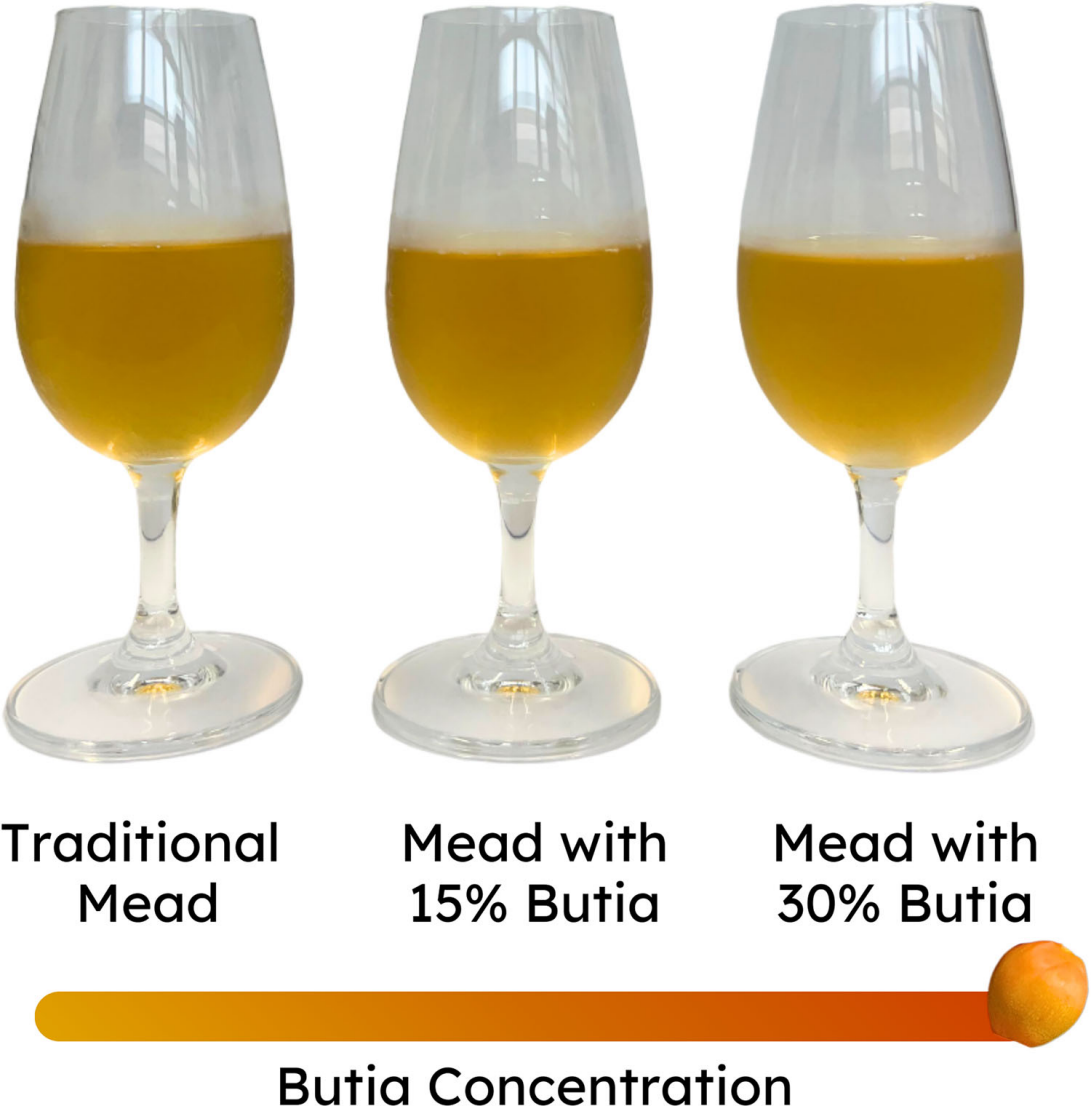
Mead matured with different concentrations of *Butia catarinensis*

The instrumental color of the meads showed significant differences (p < 0.05) for the CIELab values, except for the hue angle (Table 3). Regarding lightness (L*), all samples presented intermediate values, with a tendency toward lighter shades and a yellow color (positive b* value). Additionally, the samples showed positive a* values, indicating a tendency toward red color, particularly for the samples matured with butia. This result may be due to the transfer of pigments from the butia peel to the BM15 and BM30 samples (Figure 3). Through analysis of the hue angle, it was observed that the samples predominantly exhibited a yellowish color. The chroma (C*) values indicate that the sample with the highest butia concentration (BM30) presented a more intense and saturated color (*p* < 0.05). The combination of parameters indicates an orange‐yellow color of the beverages, with moderate intensity and saturation. Despite the significant differences observed in these parameters, the Δ*E* values (a parameter indicating of color difference between samples) when comparing TM with BM15 and BM30 were 0.61 and 1.52, respectively, both lower than the threshold for perceivable color differences by visual inspection (Δ*E* ≥ 2) (M. Li et al., 2024; Martin, 2015).

**TABLE 3:** Instrumental CIELab color of different meads.

Samples	L*	a*	b*	C*	h
**TM**	60.80 ± 0.05^a^	1.24 ± 0.04^b^	38.61 ± 0.04^a^	37.61 ± 0.13^b^	87.80 ± 0.14^a^
**BM15**	60.42 ± 0.24^b^	1.44 ± 0.01^a^	38.13 ± 0.04^b^	37.37 ± 0.15^b^	87.86 ± 0.05^a^
**BM30**	59.94 ± 0.05^c^	1.39 ± 0.01^a^	37.36 ± 0.15^c^	38.38 ± 0.02^a^	87.97 ± 0.05^a^

Results expressed as mean ± standard deviation.

Different letters in the same column indicate significant differences (*p* < 0.05) between samples according to Tukey's test.

TM: traditional mead; BM15: mead matured with 15% butia; BM30: mead matured with 30% butia.

### 3.4 Profile of Volatile Organic Compounds (VOCs)

The volatile profile is a key determinant of the sensory identity of mead, with contributions from the honey, the metabolic activity of yeast during fermentation, and the addition of fruits during maturation (Chitarrini et al., 2020; Li and Sun, 2019; Reitenbach et al., 2025; Webster et al., 2025). In this study, 17 volatile organic compounds were identified and quantified in the traditional mead (TM) and butia‐matured meads (BM15 and BM30), including esters (8), alcohols (4), acids (3), terpenes (1), and ketones (1) (Table 4), which are consistent with the main classes reported in other mead studies (Chitarrini et al., 2020; Li and Sun, 2019; Romano et al., 2021; Santos da Silva et al., 2025). The most notable finding was a clear qualitative differentiation of the profiles. For statistical comparison of quantitative concentrations, values below the limit of detection (LOD) were imputed as LOD/2 for ANOVA (Ibáñez‐Vea et al., 2011; Chang et al., 2016). For compounds that were detected and quantified in more than one treatment, no significant differences in their concentrations were found (*p* > 0.05). This finding contrasts with mead studies where fruits were added during active fermentation, which reported significant quantitative changes (Chitarrini et al., 2020; Santos da Silva et al., 2025). This divergence highlights the fundamental distinction between the two processes: fruit addition during fermentation leads to the biotransformation of substrates and the generation of new volatile compounds via yeast metabolism, whereas post‐fermentation maturation primarily facilitates the infusion of pre‐existing aromas from the fruit into the beverage (Reitenbach et al., 2025; Webster et al., 2025).

**TABLE 4:** Profile of volatile organic compounds in the traditional and butia‐matured meads.

Volatile compound concentration (µg/L)								
N°	Compound	n°CAS	RI exp.	RI lit.	TM	BM15	BM30	Odour description
**Esters**								
1	Phenethyl acetate	103‐45‐7	1792	1791	66.98 ± 0.12	<25.00*	<25.00*	Rose, honey, tobacco
2	Ethyl decanoate	110‐38‐3	1627	1633	<25.00*	<25.00*	66.77 ± 0.06	Grape, pear
3	Ethyl dodecanoate	106‐33‐2	1843	1849	<25.00*	66.71 ± 0.11	<25.00*	Floral, fruity, leaf
4	Ethyl 9‐hexadecenoate	54546‐22‐4	2267	2267	66.81 ± 0.12	66.66 ± 0.02	66.65 ± 0.04	—
5	Ethyl hexadecanoate	628‐97‐7	2246	2246	71.65 ± 4.48	73.31 ± 1.99	72.20 ± 4.02	Wax
6	Ethyl octadecanoate	111‐61‐5	2453	2455	66.72 ± 0.07	67.07 ± 0.14	67.08 ± 0.33	—
7	Ethyl lactate	97‐64‐3	1333	1339	69.71 ± 2.56	68.89 ± 1.24	<25.00*	Floral, fruity
8	Ethyl myristate	124‐06‐1	2040	2059	<25.00*	67.25 ± 0.61	<25.00*	Wax, ether
**Alcohols**								
9	Pentyl alcohol	71‐41‐0	1205	1206	58.46 ± 5.10	45.97 ± 18.98	39.47 ± 10.97	Balsamic, yeast, fruity
10	Tetradecyl alcohol	112‐72‐1	2164	2174	61.53 ± 0.04	61.51 ± 0.01	61.41 ± 0.01	Coconut
11	Ethyl oleate	111‐62‐6	2476	2476	<25.00*	66.72 ± 0.02	66.71 ± 0.08	Dairy
12	Phenethyl alcohol	60‐12‐8	1893	1890	13.76 ± 0.36	14.62 ± 5.33	12.78 ± 4.81	Honey, fruity, wine
**Acids**								
13	Acetic acid	64‐19‐7	1440	1440	68.84 ± 1.84	79.60 ± 13.45	74.70 ± 0.01	Sour, vinegar
14	Hexanoic acid	142‐62‐1	1845	1845	68.16 ± 0.20	69.86 ± 2.48	<25.00*	Cheese, sour
15	Octanoic acid	124‐07‐2	2052	2052	56.26 ± 1.46	54.99 ± 0.66	55.68 ± 1.17	Cheese, grass, oil
**Terpenes**								
16	Linalool oxide	5989‐33‐3	1430	1429	7.12 ± 0.89	6.52 ± 0.15	6.51 ± 0.19	Floral
**Ketones**								
17	Acetoin	513‐86‐0	1269	1268	20.34 ± 2.98	26.96 ± 7.85	<25.00*	Butter, cream

Means ± standard deviation (*n* = 3).

n°CAS: chemical abstract service. RI exp: experimental retention index. RI lit: retention index according to the literature (https://webbook.nist. gov/). *Limit of detection (LOD). For statistical comparison (ANOVA), samples with concentrations below the LOD were assigned a value of LOD/2. TM: traditional mead; BM15: mead matured with 15% butia; BM30: mead matured with 30% butia. Odour description: https://www.flavornet.org/flavornet.html and https://www.femaflavor.org/flavor‐library/.

Notable qualitative distinctions were observed in the volatile profile of meads, particularly among key esters. Phenethyl acetate (characterized by rose, honey, and tobacco aromas), for example, was detected exclusively in the traditional mead sample (66.98 µg/L). Similarly, Santos da Silva et al. (2025), who evaluated the volatile compounds in traditional and genipap meads, found that the concentration of this compound was approximately five times higher in traditional meads than in fruit meads. This pattern indicates that phenethyl acetate is primarily associated with the fermentation of honey and its presence can be diminished or masked by the addition of fruits. Other compounds, including phenethyl alcohol (honey, fruity, and wine aroma), ethyl hexadecanoate (wax), ethyl octadecenoate, pentyl alcohol (Balsamic, yeast, fruity) and linalool oxide (Floral) were consistently detected in all samples. Most of the compounds found in this study, with predominance of esters, are known to contribute to the fruity and floral notes characteristic of mead and other fermented beverages, related to their fundamental origin from yeast metabolism during fermentation (Kuś et al., 2022; Li and Sun, 2019; Pascoal et al., 2019; Starowicz and Granvogl, 2022; Webster et al., 2025). Furthermore, these same compounds were also reported by Santos da Silva et al. (2025) in both traditional and genipap meads, although at higher concentrations than those found in the present study.

A critical observation in the mead VOCs was the absence of key volatile compounds typically reported in other *Butia* species. The volatile profile of mature *Butia capitata* fruit is dominated by ethyl hexanoate (65.9%), ethyl butanoate (14.5%), and methyl hexanoate (9.64%) (Aguiar et al., 2014), while ethyl hexanoate and 3‐methyl‐1‐butanol were identified as major volatiles in fermented *Butia odorata* wine (Bernardi et al., 2014). The fact that none of these characteristic compounds were detected in the meads matured with *B. catarinensis* leads to two non‐mutually exclusive explanations: (i) *B. catarinensis* possesses an intrinsically different volatile profile, producing a distinct set of aroma compounds compared to its congeners; and/or (ii) the post‐fermentation maturation process in a low‐ethanol mead (∼10% ABV) and low temperature (4°C) was ineffective in extracting these specific non‐polar odorants, which are highly soluble in high‐ethanol spirits like cachaça (∼38% ABV). In contrast, butia maturation led to the exclusive presence of some specific esters in the mead. Ethyl decanoate (grape and pear aroma) was identified only in the BM30 sample (66.77 µg/L), while ethyl dodecanoate (floral and fruity aroma) was detected only in BM15 (66.71 µg/L). The presence of ethyl decanoate aligns with the volatile profile of the fruit during its post‐harvest phase. In *Butia capitata*, this compound was detected only after 3–6 days of storage and was absent in freshly harvested mature fruit (Aguiar et al., 2014), suggesting that this compound is related to a post maturation process of butia fruits. While the volatile profile showed primarily qualitative rather than quantitative changes, these subtle modifications in aromatic composition provide important context for understanding the distinct sensory characteristics of the butia‐matured meads.

### 3.5 Sensory Analysis

**FIGURE 4:**
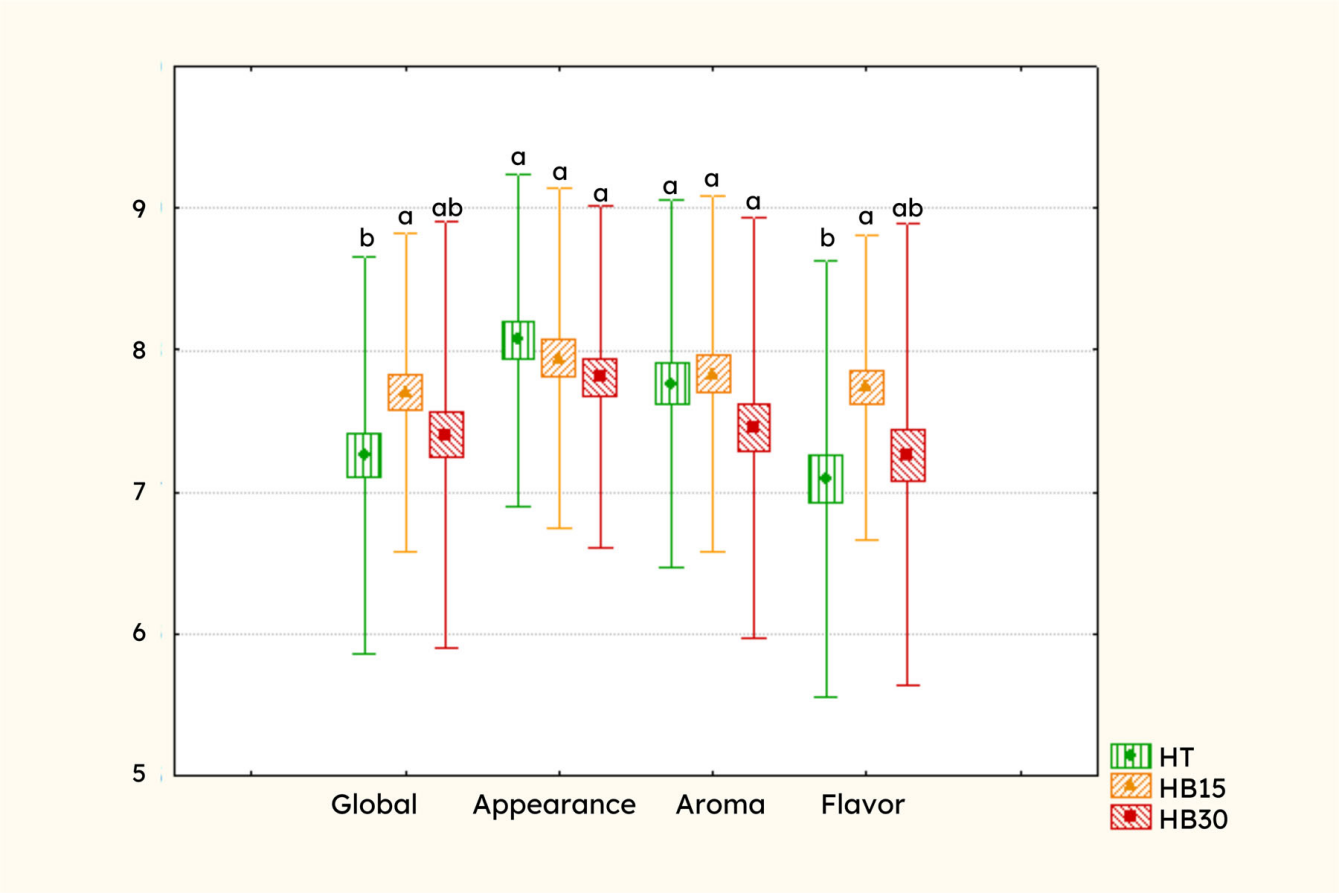
Overall and attribute‐specific liking of meads matured with different concentrations of butia. Different letters in the same attribute indicate significant differences (*p* < 0.05) between samples by the Tukey test. TM: traditional mead; BM15: mead matured with 15% butia; BM30: Mead matured with 30% butia. Scores on the 9‐point hedonic scale: (1) extremely disliked (2) moderately disliked (3) regularly disliked (4) slightly disliked (5) neither liked nor disliked (6) slightly liked (7) regularly liked (8) moderately liked (9) extremely liked.

The mean scores for overall and attribute‐specific liking of the meads are presented in Figure 4. BM15 sample achieved the highest ratings for overall liking (7.75) and flavor (7.80), with higher scores compared to the traditional mead (*p* < 0.05). No significant differences (*p* < 0.05) were observed among samples regarding aroma and appearance. The overall acceptance index of the three formulations ranged from 81.11% to 86.11%, demonstrating a high level of consumer approval (acceptable threshold ≥70%) (Dutcosky, 2013).

**FIGURE 5:**
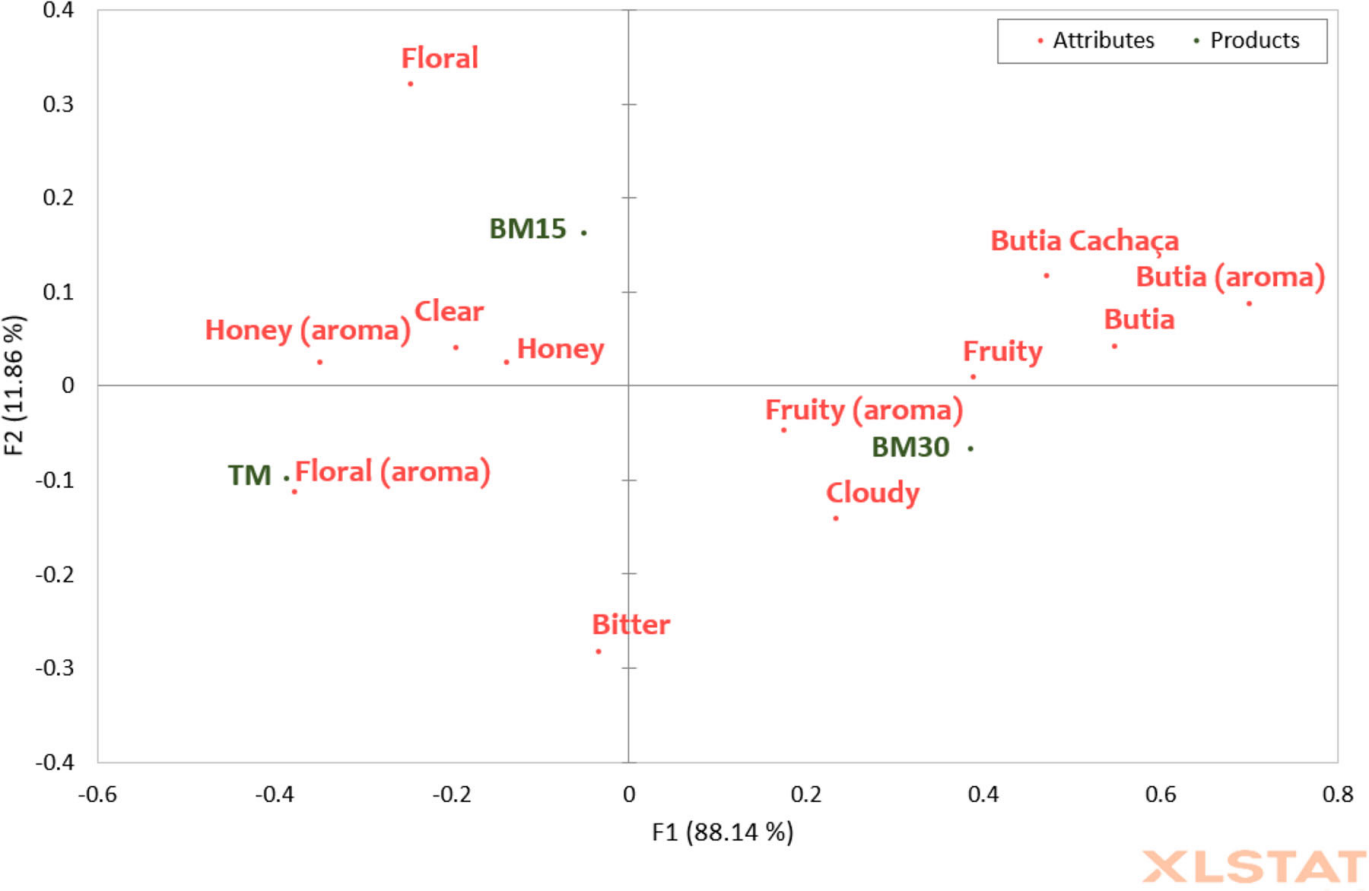
Correspondence analysis using the sensory attributes employed to describe the mead samples in the check‐all‐that‐apply (CATA) test. TM: traditional mead; BM15: mead matured with 15% butia; BM30: mead matured with 30% butia.

Chitarrini et al. (2020) evaluated the effect of adding black currant fruits to mead, obtaining an overall acceptance index between 64% and 68%, with no significant differences between the fruit and traditional meads. On the other hand, Schwarz et al. (2022) found that meads produced with grape juice had higher consumer liking, being considered more balanced compared to traditional mead without fruits. Kawa‐Rygielska et al. (2019) also reported that traditional mead had a lower flavor liking score compared to meads containing grape seeds and chokeberry syrup. Based on the literature, it is noticeable that the type of fruit added to mead is a factor that can have a significant influence on the product's liking. Another crucial factor that impacts the characteristics and liking of the final product is the concentration of fruit added to the beverage. Although several studies have evaluated the influence of adding different ingredients to mead, there is still limited research on testing different fruit concentrations (Amorim et al., 2018; Schwarz et al., 2022) and performing descriptive sensory analysis (Starowicz and Granvogl, 2022) or liking tests (Chitarrini et al., 2020; Gomes et al., 2015; Kawa‐Rygielska et al., 2019) on the products developed. These are important and recommended factors when using unconventional raw materials for the development of new products, in order to identify their attributes and acceptance. Another important factor is to identify the sensory attributes of the products. For this, the results of the CATA test were analyzed through correspondence analysis (Figure 5) and the Sheskin multiple comparisons test (Table 5).

**TABLE 5:** Selection frequency (%) of attributes for which a significant difference (*p* < 0.05) was found between samples, using the Cochran's *Q* test.

Category	Attributes	TM	BM15	BM30
**Appearance**	Clear	49.00^a^	48.00^a^	34.30^b^
	Cloudy	15.70^b^	15.70^b^	29.40^a^
**Aroma**	Honey	73.50^a^	73.50^a^	59.80^b^
	Floral	26.50^a^	16.70^ab^	11.80^b^
	Fruity	20.60^b^	24.50^ab^	35.30^a^
	Butia	1.00^c^	15.70^b^	29.40^a^
**Flavor**	Honey	55.90^a^	47.10^a^	25.50^b^
	Floral	13.70^ab^	21.60^a^	7.80^b^
	Fruity	11.80^b^	23.50^b^	38.20^a^
	Butia	4.90^c^	17.60^b^	31.40^a^
	Butia Cachaça	2.90^b^	10.80^ab^	15.70^a^
	Bitter	26.50^a^	13.70^b^	26.50^a^

Different letters in the same row indicate significant differences (*p* < 0.05) between samples by Sheskin's critical difference test for multiple pairwise comparisons.

TM: traditional mead; BM15: mead matured with 15% butia; BM30: mead matured with 30% butia.

The CATA results clearly delineated the sensory profiles of the three meads. The TM sample was associated with a clear appearance, floral aroma, and honey aroma/flavor. The mead with the highest concentration of butia (BM30) was strongly defined by a pronounced fruity and butia‐driven identity, related to the attributes cloudy, fruity aroma, butia aroma, and fruity, butia, and butia cachaça flavors. The BM15 sample stood out with intermediate characteristics, exhibiting the presence of fruity, floral, and butia aromas, and floral, honey, butia, and butia cachaça flavors, along with a clear appearance. This balanced profile of BM15, which successfully integrated the novelty of the fruit without overwhelming the fundamental honey character, provides a clear sensory explanation for its superior overall liking.

**FIGURE 6:**
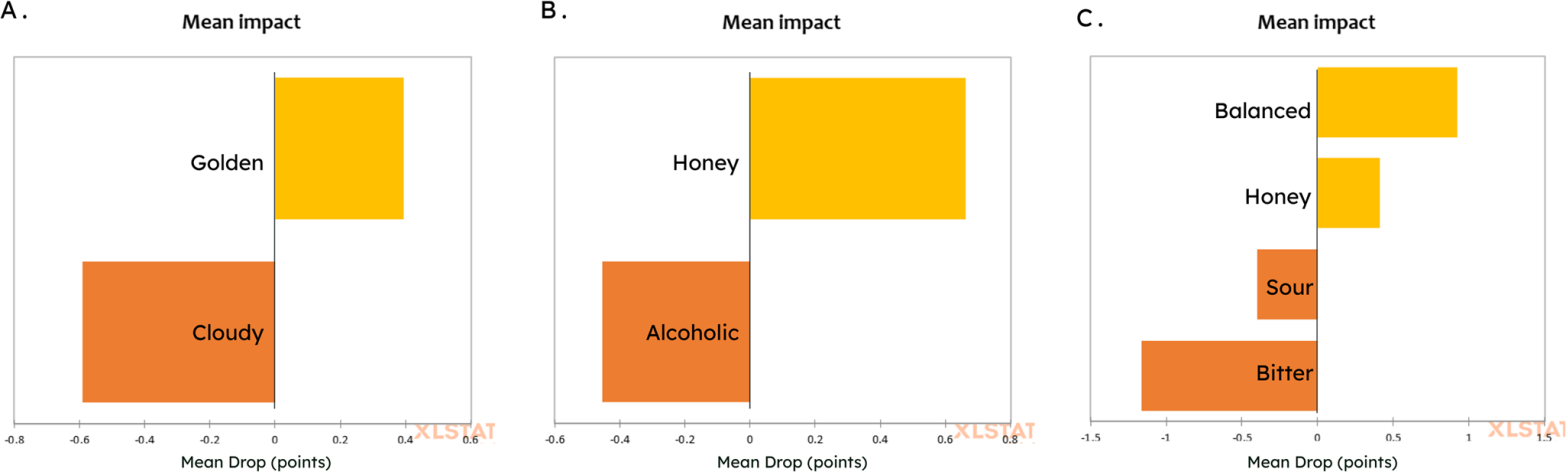
Penalty analysis of sensory characteristics (CATA) and attribute acceptance based on the meads’ evaluation by participants. (A) Average impact of appearance; (B) Average impact of aroma; (C) Average impact of flavor. Note: Penalty analysis performed in XLStat only with attributes that had a significant impact on beverage acceptance.

The sensory characteristics of mead are influenced by various factors such as the type of honey used in fermentation, the sweetness level of the beverage, the yeast strain used, and the addition of fruits or spices (Li and Sun, 2019). Schwarz et al. (2022) found that the addition of fruit (grape juice) in mead resulted in a beverage with moderate aromatic intensity, with fruity, floral, and honey aromas. Fermented beverages based on *Butia odorata* also presented floral and fruity aromatic compounds (Bernardi et al., 2014). The distinct “fruity” and “butia” aromatic attributes perceived in the BM15 and BM30 samples can be attributed to the unique qualitative ester profile revealed by GC‐MS (e.g. ethyl decanoate and ethyl dodecanoate), thereby establishing a direct link between the instrumental and sensory data. However, the sample that exhibited the strongest floral aroma was the traditional mead, and this association was likely related to the sensory prevalence of wildflower honey in this sample, as multifloral honeys contain volatile compounds characterized by floral aromas (Kang et al., 2023). The significant differences in the flavor characteristics between the samples, where participants clearly identified the presence of the fruit, are also consistent with the distinct organic acid profiles (Figure 2), which are known to directly influence the taste perception of acidity and fruitiness (Chitarrini et al., 2020). To determine which characteristics of the samples positively or negatively affected product acceptance by consumers, a penalty analysis was applied using the CATA test terms and the scores from the attribute liking test (Figure 6).

Penalty analysis (Figure 6) provided crucial insights into the drivers of liking and disliking for the meads. The values indicated which characteristics influenced the participants to like or dislike the samples. It was observed that participants appreciated the attributes golden color, honey aroma, and balanced and honey flavors in the developed products. Among the characteristics that led participants to dislike the samples, the attributes turbid, alcoholic aroma, and sour and bitter taste were the most mentioned. Therefore, the penalty analysis conclusively demonstrates that BM15's optimal acceptance was a result of its successful balance of positive drivers (golden color, honey aroma, balanced flavor) while minimizing negative ones (cloudy appearance, alcoholic aroma, sour/bitter taste). Li and Sun (2019) also observed that honey aroma was a positive aspect in the sensory evaluation of meads made with different types of honey. Previous studies that conducted quantitative descriptive analysis of commercial meads in the United States found that cloying and viscous mouthfeel were among the main descriptors for the products (Senn et al., 2021). In the present study, both the “honey” and “balanced” attributes were highlighted positively, showing the importance of sweetness balance so the beverage does not become cloying but still maintains the honey flavor.

Beyond their sensory and technological potential, sustainability considerations are particularly relevant when working with native fruits such as *Butia catarinensis*, since butia‐based products contribute to local socioecological systems (Pedroso et al., 2023). Recent studies show that the extraction and commercialization of butia fruits represent the main source of income for up to 45% of harvesters and a complementary income for the remaining 55% in traditional communities of southern Brazil (Werner‐Martins and Freitas, 2023). Because *B. catarinensis* is classified as an endangered species, value‐added products such as fermented beverages can strengthen local economies while reinforcing conservation‐through‐use practices. This connection between product development, cultural heritage, and environmental governance highlights how incorporating butia into innovative beverages may generate indirect yet meaningful sustainability impacts.

Although the results obtained in this study provide valuable insights into the chemical and sensory characteristics of traditional and butia‐matured meads, some limitations should be recognized. The maturation period was 30 days at 4°C, which may not reflect the optimal maturation condition for the beverage, considering that different maturation conditions could influence the sensory and chemical attributes of the product in distinct ways. Additionally, using only three concentrations of butia may not cover the full potential of the fruit and its variations, and it would be interesting to explore a wider range of concentrations or even different combinations with other native Brazilian fruits. Another limitation of the study was the use of only one sensory descriptive method (CATA), which, although providing valuable information on consumer perceptions, did not capture the intensity of the attributes. Future research could incorporate methods that assess the intensity of sensory attributes, such as rate‐all‐that‐apply (RATA), to obtain a more detailed evaluation. Additionally, it would be relevant to evaluate the effects of other types of honey or combinations with other ingredients, such as spices or native herbs, to expand formulation possibilities and explore mead's versatility.

## 4 Conclusions

This study demonstrated that the maturation of mead with *Butia catarinensis* significantly influences its chemical and sensory characteristics. The addition of the fruit led to marked changes in the organic acid profile, particularly an increase in malic and citric acids, and contributed to a reduction in residual sugars. Furthermore, the maturation with butia differentiated the volatile profile from the traditional mead, introducing distinct compounds like ethyl decanoate and ethyl dodecanoate, which are associated with fruity aromas. This distinct chemical and volatile profile directly influenced the sensory profile of the meads: the traditional mead (TM) was characterized by honey and floral notes, while the 30% butia mead (BM30) exhibited intense fruity and butia attributes. Most importantly, the sample with 15% butia (BM15) achieved the highest consumer acceptance by reaching an optimal balance between the distinctive fruit character and the fundamental honey notes of the traditional mead. The application of the check‐all‐that‐apply method proved to be an effective tool for sensory characterization of fruit‐meads, enabling the identification of key attributes perceived by consumers. The study demonstrated the potential of using underutilized native Brazilian fruits to innovate in fermented beverage development, while also supporting sustainable ingredient sourcing and the valorization of regional biodiversity. Based on these findings, future research should prioritize the identification of the key ‘butia’ aroma compounds using gas chromatography‐olfactometry (GC‐O) to move from a descriptive to a mechanistic understanding of the sensory profile. Additionally, it is recommended to use intensity‐based sensory methods to quantify the ideal intensity of the key descriptors identified.

## Author Contributions

**Rodrigo Ribeiro Arnt Sant'ana**: conceptualization, visualization, methodology, validation, formal analysis, writing – original draft, investigation, writing – review and editing. **Ana Letícia Andrade Ferreira**: data curation, formal analysis, writing – original draft. **Rafaela Diogo Silveira**: formal analysis, data curation. **Juliane Elisa Welke**: formal analysis, data curation. **Bruna Rafaela da Silva Monteiro Wanderley**: formal analysis, data curation, writing – original draft. **Luciano Valdemiro Gonzaga**: formal analysis. **Adriane Costa dos Santos**: formal analysis. **Ana Carolina Oliveira Costa**: formal analysis, writing – review and editing, methodology. **Renata Dias de Mello Castanho Amboni**: writing – review and editing, resources. **Carlise Beddin Fritzen‐freire**: conceptualization, visualization, methodology, writing – review and editing, resources, funding acquisition, supervision.

## Funding

The authors are grateful to CAPES (Coordenação de Aperfeiçoamento de Pessoal de Nível Superior) ‐ Finance code 001, CNPq (Conselho Nacional de Desenvolvimento Científico e Tecnológico, n.140616/2021‐7) and FAPESC (Fundação de Amparo à Pesquisa e Inovação do Estado de Santa Catarina ‐ code 2021TR000353) for their research support; to Néctar Hidromel Bebidas LTDA. for supplying the raw materials for the research. RD was granted a fellowship (PQ1D) from CNPq (Grant number 305007/2022‐0).

## Conflicts of Interest

The authors declare the existence of conflicts of interest, since the author Rodrigo Ribeiro Arnt Sant'Ana is a partner in a micro meadery. This fact did not influence the data and results presented in this manuscript.
